# Limited access to HIV prevention in French prisons (ANRS PRI^2^DE): implications for public health and drug policy

**DOI:** 10.1186/1471-2458-11-400

**Published:** 2011-05-27

**Authors:** Laurent Michel, Marie Jauffret-Roustide, Jerôme Blanche, Olivier Maguet, Christine Calderon, Julien Cohen, Patrizia M Carrieri

**Affiliations:** 1 Health and Medical Research National Institute, Research Unit 669, Paris, France; 2 Univ Paris-Sud and Univ Paris Descartes, UMR-S0669, Paris, France; 3 AP-HP, Emile Roux Hospital, Centre de Traitement des Addictions, Limeil-Brévannes, France; 4 Infectious Diseases Department, National Institute for Public Health Surveillance, Saint-Maurice, France; 5 CERMES3, UMR CNRS 8211, University Paris Descartes, Inserm U988, Paris, France; 6 INSERM, U912 Economic & Social Sciences, Health Systems & Societies, Marseille, France; 7 IRD, Aix Marseille Université, Faculté de Médecine, UMR-S912, Marseille, France; 8 Southeastern Health Regional Observatory (ORS-PACA), Marseille, France; 9 CCMO Conseil, 3 rue des déportés, 28150 Ymonville, France

## Abstract

**Background:**

Overpopulation, poor hygiene and disease prevention conditions in prisons are major structural determinants of increased infectious risk within prison settings but evidence-based national and WHO guidelines provide clear indications on how to reduce this risk. We sought to estimate the level of infectious risk by measuring how French prisons adhere to national and WHO guidelines.

**Methods:**

A nationwide survey targeting the heads of medical (all French prisons) and psychiatric (26 French prisons) units was conducted using a postal questionnaire and a phone interview mainly focusing on access to prevention interventions, i.e. bleach, opioid substitution treatment (OST), HBV vaccination and post-exposure prophylaxis (PEP) for French prisoners. Two scores were built reflecting adherence to national and WHO international guidelines, ranging from 0 (no adherence) to 10 (maximum adherence) and 0 to 9 respectively.

**Results:**

A majority (N = 113 (66%)) of the 171 prisons answered the questionnaires, representing 74% coverage (46,786 prisoners) of the French prison population: 108 were medical units and 12 were psychiatric units. Inmate access to prevention was poor. The median[IQR] score measuring adherence to national guidelines was quite low (4.5[2.5; 5.5]) but adherence to WHO guidelines was even lower 2.5[1.5; 3.5]; PEP was absent despite reported risky practices. Unsuitable OST delivery practices were frequently observed.

**Conclusions:**

A wide gap exists between HIV prevention policies and their application in prisons. Similar assessments in other countries may be needed to guide a global policy reform in prison settings. Adequate funding together with innovative interventions able to remove structural and ideological barriers to HIV prevention are now needed to motivate those in charge of prison health, to improve their working environment and to relieve French prisoners from their currently debilitating conditions.

## Background

HIV prevention and risk reduction interventions in prison settings are a major public health concern and a critical political issue [1]. Infectious diseases are more prevalent in prison than in the general population and many reports indicate that prison stay is an independent risk factor for the transmission of blood-borne viruses' [2-4]. Risky behaviors are frequent while the dramatic prevalence of psychiatric disorders and the prison context (lack of hygiene, promiscuity, sexual violence, overpopulation and violence) exacerbate the risks [4-7]. Many inmates cycle in and out of prison repeatedly, increasing the likelihood that any [4] infections contracted in prison could soon affect the general community [2,8].

In France, the prison population has increased by one third in the last 10 years, partly due to the criminalization of drug use. In 2008, 14.3% of sentences were for drug-related offences, 36% of which involved prison sentences [9].

The Ministry of Health has been responsible for health in French prisons since 1994. National guidelines [10] recommend equity in access to prevention measures and continuity of care between the community and prisons and back to the community. However, the question of access to prevention measures for HIV and other infectious diseases is not referred to in any great detail in these guidelines. Such access is referred to in a 1996 Ministry of Health/Ministry of Justice joint circular [11] regulating HIV prevention in prison settings in which several harm reduction (HR) measures available in the general community (including needle syringe exchange programs) are not permitted.

The main objective of this national survey is to estimate the level of infectious risk by measuring to what extent French prisons adhere to international guidelines for HIV prevention in prison and to national policies, as regulated by the 1996 circular [11] and national guidelines [10]. A secondary objective is to identify which particular characteristics of prison settings predict non-adherence to national and WHO guidelines.

## Methods

A nationwide survey targeting all French prisons was conducted between November 2009 and May 2010. A questionnaire focusing primarily on access to infectious disease prevention and HR measures for French prisoners was first sent to the heads of medical (all prisons) and psychiatric (26 prisons) units (see additional file 1: ANRS-PRI^2 ^DE Inventory Questionnaire). Additional detailed information about issues regarding access to HIV prevention was gathered through a structured phone interview. More specifically the questionnaire collected data about characteristics of the prisons with 10 sections, accounting for a total of 46 items, each exploring access to a specific prevention measure.

We defined "international guidelines for HIV prevention in prison" as those recommendations provided by WHO, in collaboration with UNAIDS and UNODC in their document entitled "Effectiveness of interventions to address HIV in prisons" which includes interventions for preventing not only HIV but other infectious diseases in prison settings [4]. This WHO document was chosen from among several other tools and reports as it is the most comprehensive document on HIV prevention in prison settings.

Two scores of adherence to national and international guidelines were built in order to both evaluate the implementation of HIV prevention and other HR measures in French prisons and to estimate the level of infectious risk. Table 1 reports items corresponding to national and international guidelines and how adherence to each specific recommendation was scored. Finally, two global scores were built, for national and international guidelines respectively, by summing the subscores corresponding to each recommendation. Each subscore was dichotomous (0 = non- adherence, 1 = adherence) with the exception of the scores for opioid substitution treatment and access to condoms. The corresponding scores for these latter items varied between 0 and 2 in order to give them a higher weighting, as their effectiveness in HIV prevention has already been established [4].

**Table 1:** Scoring method for computing adherence to national and WHO guidelines in French prisons (ANRS PRI^2^DE)

	French Guidelines*	Score	WHO Guidelines**	Score
**Information-Education-Communication**	• Distribution of information, Flyers or other tools on HIV, Hepatitis and IST prevention at prison entry • **AND**Harm reduction, HIV, sexuality and hepatitis education programs in prison settings	**1**	• Availability of Information/education at entry or during prison stay • Peer education programs available • **AND**availability of clean injecting equipment + condoms **(0 if not)**	0.5 0.5 **1**

**Testing - Counseling**	• Systematic HIV, HBV and HV testing proposed at prison entry (RC) and during prison stay (all prisons) • **AND**systematic negative test return	**1**	• Testing for HIV, HBV, HCV systematically proposed at entry (RC) and during prison stay (all prisons) • **AND**availability of clean injecting equipment + condoms **(0 if not)**	**1**

**Condoms - Lubricants**	• Available information on condoms and lubricant access • Male condoms and lubricants accessible and female condoms accessible for prisons with female prisoners, • Condoms accessible in a place other than the medical unit	**2 **if 3 items **1 **if only 2 items **0 **if 1 or 0 item	• Condoms available in various locations • Water-based lubricants available • Male condoms and lubricants accessible and female condoms accessible for prisons with female prisoners	1 0.5 0.5 **2**

**Opioid Substitution Therapy**	• Induction at entry (RC) + induction during prison stay + continuity of OST at entry (all prisons)	1	• Induction at entry (RC) + induction during prison stay + continuity of OST at entry (all prisons)	1
	• No ceiling dosage	0.5	• No ceiling dosage	0.5
	• No BHD crushing or dilution	0.5 **2**	• No BHD crushing or dilution	0.5 **2**

**Bleach**	• Existing and intelligible information on the use of bleach in harm reduction for all prisoners • **AND**Bleach renewal at least every 2 weeks	**1**	• At least 2 locations/access for bleach inside prison (penitentiary distribution, purchasable inside prison, available in medical unit) • **AND**Intelligible information for HR purpose accessible for all prisoners	**1**

**HBV Vaccination**	• Systematic HBV vaccination proposal for all seronegative prisoners	**1**	*Not applicable*	

**Post-Exposition Prophylaxis**	• All prisoners informed of the PEP availability inside prison	**1**	• All prisoners informed of the PEP availability inside prison	**1**

**Hair cutting procedures/protocols**	• Existing hair cutting disposal or protocol	**1**	*Not applicable*	

**Needle Exchange Programs**	*Not applicable*		• NEP are available	**1**

**TOTAL**		**10**		**9**

* References are the1996 French Ministry of Health/Ministry of Justice joint circular and the 2004 Ministry of Health/Justice National Guidelines

**Reference is the 2007 WHO report untitled "Effectiveness of interventions to address HIV in prisons. Evidence for action technical papers. Geneva, WHO-UNODC-UNAIDS"

Potential score ranges for measuring adherence were as follows: 0 to 10 for national guidelines and 0 to 9 for international guidelines. For each subscore, we computed the proportion of prisons adherent to national and WHO guidelines as well as their 95% confidence intervals.

A linear regression model was used to assess the relationship between each prison characteristic and the level of adherence to national and WHO guidelines.

The National Agency for Research on Aids and Viral Hepatitis (ANRS) committee granted approval for the project. As only aggregated and openly available data were used in the study, authorization from the *Commission Nationale Informatique et Liberté *was not required.

### Study group

A majority (N = 113 (66%)) of the 171 prisons answered the questionnaires, representing 74% (46 786 prisoners) of the French prison population: 108 (63%) medical units and 12 (46%) psychiatric units. Finally, 103 prisons having complete data for all the items used for assessing adherence to national and WHO guidelines were included.

## Results

No significant difference was found between the structural characteristics of prisons included in the study and those excluded.

Among the 103 selected prisons, covering 43 365 incarcerated subjects (69%), 62% are remand centers (RC), 13% are both RC and prisons for persons sentenced (PPS), and 25% are PPS only (including 5 security prisons). Seventy four (72%) are male only prisons, 1 is a female only prison, and 28 are mixed male and female prisons. Among the 103 prisons, 24% receive juvenile offenders. The mean number of prisoners per prison is 421, ranging from 36 to 3785.

### Descriptive results

Table 2 shows, for each subscore, the proportion of prisons adherent to national and/or WHO guidelines.

**Table 2:** Proportion of prisons adherent to national and WHO guidelines for each sub-score composing the global adherence score (ANRS PRI^2^DE) (N = 103 prisons)

	France % [95%CI]	WHO % [95%CI]
Bleach: access and information	14 [7-20]	6 [1-10]

Condom & Lubricants: access and information	9 [3-14]	12 [5-18]

Opioid Substitution Treatment	27 [18-36]	27 [18-36]

HIV-HCV-HBV Screening	64 [55-74]	0%*

HBV vaccination	83 [75-90]	**NA**

Information Education Communication	66 [57-75]	0%*

Post-Exposition Prophylaxis	23 [14-31]	23 [14-31]

Hair cutting measures	33 [24-42]	**NA**

NSP	**NA**	0%**

* A condition of simultaneous availability of condoms and NSP (not authorized in French prisons) is required in WHO guidelines

** NSP are not authorized in French prisons but are available in the community. NA = Not Available

#### Bleach

Information about how to use bleach for HR purposes is considered as accessible and intelligible for prisoners in only 22% of prisons. Bleach distribution is absent in 10% of prisons.

Prisons adherent to national or WHO bleach guidelines account for 14% and 6% respectively (Table 2).

#### Condoms

Male condoms are available in nearly all prisons (95%). Female condoms are available in only 21% of prisons. Lubricants are distributed with condoms in only 51% of prisons. When available (n = 99), condoms are most often available only in medical units (96 prisons). In 20% of prisons they are also available in a different location (most often external associations' rooms, libraries or visiting rooms). According to medical staff, prisoners have knowledge of the availability of condoms and how to access this availability in 73% of prisons.

National and WHO guidelines for condom access are adhered to in 9% and 12% of prisons respectively (Table 2).

#### Opioid Substitution Therapy (OST)

In the 103 prisons, 3854 prisoners (9%) receive OST, 2607 buprenorphine (6%) and 1247 methadone (3%). OST coverage among inmates ranges from 0% to 40%.

Medical staffs declare that they do not, or do not systematically renew the prescription of OST for prisoners at entry in 11% of prisons, while in 13% OST is never initiated at entry nor during incarceration; 23% never initiate buprenorphine at entry nor during detention; 23% never initiate methadone. In 17% of all prisons medical staff declare limiting the prescribed dosage of methadone, despite the lack of any officially established threshold. Nearly one fifth of prisons crush or dilute buprenorphine, three prisons applying both practices. 

Only 27% of prisons are completely adherent to both national and WHO guidelines in terms of access to OST (Table 2).

#### BBV screening

Concerning BBV screening, 91%, 90% and 89% of the heads of medical staff declare to systematically propose screening at entry for all prisoners for HIV, HBV and HCV respectively. Negative test results are systematically provided to prisoners in 70%, 64% and 65% of cases for HIV, HBV and HCV respectively. In 102 prisons, testing for HIV, HBV or HCV is possible during detention.

Seventy four percent of prisons are adherent to national guidelines while no prison is adherent to WHO guidelines (Table 2). This is because international guidelines state that BBV screening should always be accompanied by access to clean injecting equipment and condoms (Table 1).

#### HBV Vaccination

When prisoners are HBV negative, 83% medical units systematically propose vaccination to inmates, so they adhere to national guidelines on prevention (Table 2). HBV vaccination in prison settings is not mentioned in the WHO guidelines used as reference. It is however recommended, especially for injecting drug users, in different WHO reports including "Health in prisons - a WHO guide to the essentials in prison health", 2007.

#### Information, Education, Communication on BBV, STI and harm reduction

According to the heads of medical units, the distribution of flyers or other tools on HIV hepatitis and IST prevention is performed at prison entry in 86% of prisons but also during detention in 90% of prisons. HR, HIV, sexuality and hepatitis education programs provided by peers or external clinical staff (NGOs, associations or care units) are available in 77% of prisons. 

Sixty eight and no (0%) prisons adhere to national and WHO guidelines on information and education for prevention concerns respectively (Table 2).

#### PEP or care for consequences of risk practices

Medical staffs declare that prisoners have no knowledge of PEP availability in 47% of prisons and report being unable to answer PEP related questions in 31% of prisons. During the 12 months preceding this study, only 3 PEP were prescribed for prisoners (none for drug use). Thirty five (34%) heads of medical units declare providing care to prisoners for abscesses possibly due to injection practices.

 Twenty-three percent of prisons are adherent to both national and WHO guidelines (Table 2).

#### Haircutting

French guidelines also provide recommendations for prevention of blood-borne viruses (BBV) transmitted through unsafe hair-cutting procedures based on the availability of sterile material and a standardized protocol. This issue is not dealt with in WHO guidelines.

 Thirty four (33%) prisons are adherent to national hair-cutting guidelines (Table 2).

#### Needle syringe programs (NSP)

WHO guidelines underline the need to implement NSP as a major tool for HIV prevention in prison settings. However, they are not mentioned in French guidelines. Because of this, adherence to national guidelines cannot be computed and consequently no prison is adherent to WHO guidelines.

### Adherence to national and WHO guidelines

**Figure 1:**
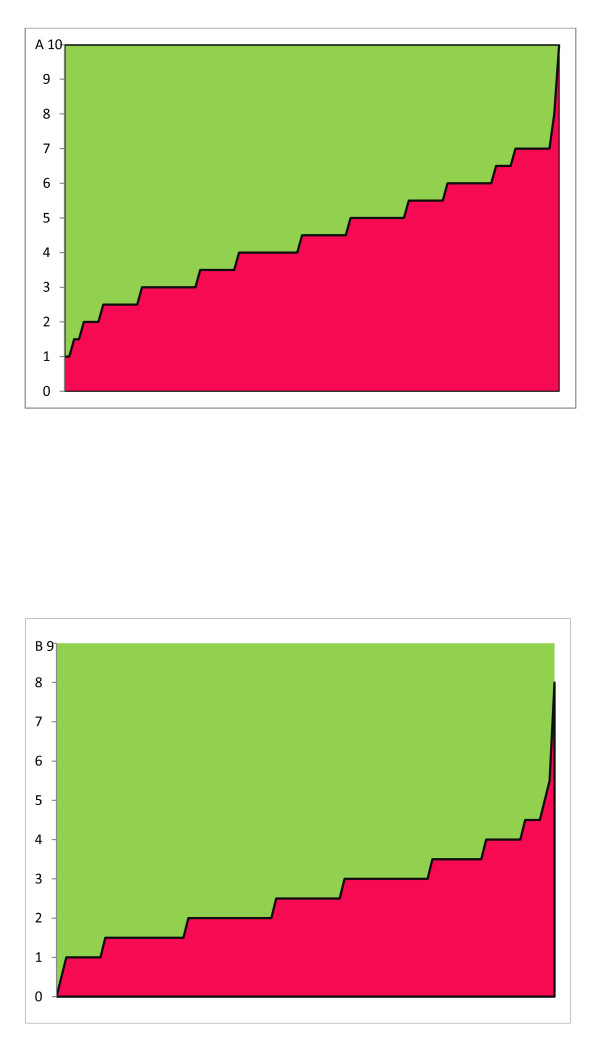
Gap (in green) between national guidelines (A), WHO guidelines (B) and practices in French prisons (N = 103) as expressed by the level of adherence score (0 = no adherence, 10 = max adherence and 0 = no adherence, 9 = max adherence, respectively).

Figures 1A and 1B show the level of adherence to national and WHO guidelines respectively.

The adherence score for national guidelines ranged between 1 to 10, median values remaining rather low (4.5[2.5; 5.5]) as Figure 1A shows. The distance between each bar and the expected adherence value highlighted a large area over the curve, providing a visual estimate of the distance between national guidelines and local practices.

As reported in Figure 1B the adherence score for WHO guidelines ranged between 0 to 9 and median values remained low (2.5[1.5; 3.5]), which confirms the gap between international guidelines and practices.

### Relationship between prison characteristics and the level of adherence to guidelines

Apart from PPS, which presented lower national adherence scores than RC, no other relationship was found between characteristics of prisons (Table 3) and the level of adherence to national or WHO guidelines.

**Table 3:** Relationship between structural prison factors and adherence to national and WHO guidelines using univariate linear regression (N = 103).

		National guidelines		WHO guidelines	
	**N (%)**	**Coefficient (95%)**	**p-value**	**Coefficient (95%)**	**p-value**

**Type of prison**:					
- RC* (ref)	77 (75)				
- PPS**	21 (20)	-1.10 (-1.89; -0.30)	0.01	0.03 (-0.51; 0.56)	0.92
- Security PPS	5 (5)	-0.64 (-2.13; 0.85)	0.40	-0.05 (-1.05; 0.96)	0.93

**Number of prisoners:**					
- < 100 (ref)	17 (17)				
- 100-350	40 (39)	-0.12 (-1.09; 0.86)	0.81	-0.25 (-0.87; 0.37)	0.43
- 350-600	23 (22)	-0.07 (-1.15; 1.00)	0.89	0.11 (-0.58; 0.80)	0.76
- > = 600	23 (22)	0.08 (-1.00; 1.16)	0.88	0.15 (-0.54; 0.84)	0.67

**Psychiatric Unit:**					
- no (ref)	85 (83)				
- yes	18 (17)	0.69 (-0.17; 1.55)	0.11	0.14 (-0.42; 0.70)	0.61

**Main practitioner gender: (n = 97)**					
- male (ref)	79 (81)				
- female	18 (19)	-0.06 (-0.92; 0.80)	0.89	-0.28 (-0.84; 0.29)	0.33

* RC: Remand Center

** PPS: Prison for Persons Sentenced

## Discussion

This study, the first conducted in France on access to HIV prevention interventions and availability of HR tools in prison settings clearly shows a wide gap between national & international policies and local practices. Adherence to national guidelines on availability of information and access to HIV prevention and HR measures is very poor, and preventive measures like NSP promoted by WHO guidelines are absent, even as local initiatives.

The lack of structural predictors of adherence suggests that practices depend on the ideology of the local administration even if they are not evidence-based.

When focusing on each specific tool for HIV prevention or HR, the heterogeneity of practices and gaps between national and international policies becomes more striking: such is the case for bleach distribution, NSP, PEP and HBV vaccination.

Bleach effectiveness as a disinfectant for inactivating HIV and particularly HCV is limited in prison settings [4]. Bleach distribution does not meet the regulations expressed by the French 1996 circular in two thirds of prisons and the information about its use as a HR tool is often absent. This may be due to the shared responsibility with the criminal justice administration regarding the distribution of information, to insufficient training in health care units but also to a lack of a comprehensive and clear HR policy in French prisons [12].

Condoms are almost always available in French prison settings but in nearly half of the cases without lubricants and in three quarters of the prisons, access is restricted to medical units where confidentiality is limited. Sexuality in prison is still a taboo because it refers to homosexuality, which is an especially stigmatized practice in prison. Although prison OST coverage is progressively scaling-up (2% of the prison population in 1998 - 2 years after the Marketing Authorization Application in France - reaching 8.9% in 2010), this is more probably a reflection of increased coverage in the French opioid dependent population. OST initiation remains problematic and the use of a ceiling dosage prescription which is not consistent with official guidelines is often observed. Buprenorphine crushing or diluting practices to reduce the risk of diversion, underscore the difficulties met by health staff when trying to balance the risk of diversion with adequate care for opioid dependence, and suggest that fear of buprenorphine diversion in prisons may represent a major barrier to its access.

BBV testing at prison entry is widely proposed but according to a previous survey of prison settings, only a small percentage of prisoners agree to be tested [12]. The proportion of inmates vaccinated against HBV on prison entry increased from 13.7% in 1997 to 31.3% in 2003, probably thanks to the HBV vaccination campaign in the general population [12]. As a history of incarceration has consistently been found to be a major predictor of HBV seropositive status [13,14], accelerated strategies based on injection at days 0, 10 and 21 which are easy to perform and remain effective, need to be urgently introduced in prison settings.

The currently poor access to and implementation of PEP interventions in prisons is unfortunate and this is one of the fields, together with HBV vaccination, which deserves major attention. Information for prisoners about the existence of such HR tools is rarely available and no such treatment is prescribed for risky injection practices.

At the international level, only a few studies on the accessibility of HIV prevention and HR measures in prison settings are available. In terms of bleach, NSPs and condoms, most associated studies are from Australia and Canada, where confidentiality or anonymity are considered as crucial points [15-18]. NSPs are available in more than 50 prisons in 12 countries worldwide, mostly in Europe [3]. In Germany, one evaluation reported only a small reduction in syringe sharing in one program, due to insufficient supply of needles and syringes, access without anonymity and inadequate provision of correct sized syringes [4]. Condoms, in theory, are widely accessible in many countries, except in the United States where they are available in less than 1% of prisons [4]. In reality, condoms are often not accessible for different reasons [19], lack of anonymity being a frequently cited example [4].

One major advantage of our survey is its capacity to investigate not only accessibility to tools for reducing the risk of infection in prison but also the general information transmitted to inmates about the availability of HR tools. It is important to underline that wide scale care assessment in prison settings is rare, because it is particularly difficult to carry out. Official authorization, funding and agreement from ethics committees are only rarely obtained [20]. A second advantage is that the study can be easily replicated in other countries for international comparisons and guide a global policy reform to improve HIV prevention and general care in prison settings.

Some limitations need to be acknowledged. Although self-reports and interviews serve as the only feasible methods to study access to prevention, they may be affected by social desirability bias. However, while the risk of over-reporting availability of preventive measures was high in our study, adherence rates were rather low, suggesting that true rates are likely to be even lower. Although no significant difference was found between the structural characteristics of prisons included in the study and those excluded, it is possible that the former could be more concerned by prevention/harm reduction issues. Accordingly, we can presume that we selected those more likely to be adherent to national or international guidelines. This means that the level of adherence to national and WHO guidelines is likely to have been overestimated, which in turn strengthens our conclusions.

Secondly, it is also possible that physicians may not have been sufficiently informed about the interventions promoted by the prison administration in which they worked, but this could only be the case for bleach distribution, whose access depends on the penitentiary administration. Although the provision of care to reduce harms from injecting practices is frequently reported, drug injection remains a taboo practice in prison settings because drug use is criminalized both in prison and in the general community. This criminalization contributes to making the introduction of NSPs useless and hindering the full implementation of initiatives for reducing harm in the injecting drug inmate population. Despite these issues, in a French survey conducted among drug users in the community, 12% reported injecting practices in prison settings [21].

The failure in the implementation of a global HR strategy in prison settings can be attributed to many causes, but the primary one is that criminal justice laws and drug prohibition still prevail over the public health objectives of equity of access to prevention and care, both in prison and in the general community.

In the last ten years, the reinforcement of the criminalization of drug use has had a twofold effect. First it constitutes a barrier to the implementation of an effective HR policy in the community, decriminalization being associated with a reduced HIV prevalence among drug users [22].

Secondly, the criminalization of drug use has contributed to overpopulation in prisons. This has not been counterbalanced by the adequate implementation of preventive measures to control the increased risk of infection due to promiscuity or by an increase in health care funds which could improve the coordination and delivery of existing HR interventions. More globally with regard to health and hygiene, prison conditions for French inmates are so deplorable that interventions to reduce harm from drug use are probably not considered as a priority for health care providers and administrative professionals working in prison settings.

On the other hand, national and international guidelines are mainly designed for HIV prevention and are rapidly becoming obsolete in terms of the emerging risk of other infectious diseases like tuberculosis, hepatitis or sexually transmissible diseases. The control of HCV in correctional settings needs implementation of additional preventive measures and/or of a HR package. In terms of health policy, it would be more valuable if national and international guidelines could anticipate policies to control emerging public health problems in correctional settings.

## Conclusions

A policy reform for HIV prevention and HR in prison settings is a priority but needs to be incorporated into a wider national health policy reform to improve the general health and quality of life of French prisoners as well as equalizing access to care and prevention in prisons and the general community. A drug policy reform decriminalizing drug use seems to be the sine-qua-non condition for replacing incarcerations for the use of drugs with improved access to prevention and care for drug dependence.

HR will not be more effective in prison settings unless innovative interventions and adequate funding are introduced to motivate prison health and administrative staff, improve their working environment and finally relieve the debilitating somatic and psychological conditions of French prisoners.

## List of abbreviations

ANRS: National Agency for Research on Aids and Viral Hepatitis; WHO: World Health Organization; OST: Opioid Substitution Treatment; HBV: Hepatitis B Virus; PEP: Post-Exposure Prophylaxis; HIV: Human Immunodeficiency Virus; HR: Harm Reduction; UNAIDS: United Nations programme on AIDS; UNODC: United Nations Office on Drugs and Crime; RC: Remand Center; PPS: Prison for Persons Sentenced; HCV: Hepatitis C Virus; IST: Infection Sexually Transmissible; NGO: Non-Governmental Organization; BBV: Blood Born Viruses; NSP: Needle Syringe Program;

## Competing interests

The authors declare that they have no competing interests.

## Authors' contributions

LM designed the study, supervised the design of the questionnaire and data collection, and wrote and revised the manuscript. MJR contributed to the design of the study and the questionnaire and revised the final version of the manuscript. OM and CC contributed to the design of the questionnaire, collected the data and contributed to the data analysis. JB and JC conducted the data analysis in and contributed to the results section. MPC contributed to the design of the study, wrote the manuscript and edited the final version. All authors read and approved the final manuscript.

## Pre-publication history

The pre-publication history for this paper can be accessed here:

http://www.biomedcentral.com/1471-2458/11/400/prepub

## Supplementary Material

Additional file 1 **ANRS-PRI^2 ^DE Inventory Questionnaire**. This file presents the questionnaire, collecting data about characteristics of the prisons with 10 sections, accounting for a total of 46 items, each exploring access to a specific prevention measure. Click here for file

## References

[B1] Fazel S Baillargeon J The health of prisoners Lancet 2010 10.1016/S0140-6736(10)61053-7 21093904

[B2] Dolan K Kite B Black E Aceijas C Stimson GV HIV in prison in low-income and middle-income countries Lancet Infect Dis 2007 7 1 32 41 10.1016/S1473-3099(06)70685-5 17182342

[B3] Jurgens R Ball A Verster A Interventions to reduce HIV transmission related to injecting drug use in prison Lancet Infect Dis 2009 9 1 57 66 10.1016/S1473-3099(08)70305-0 19095196

[B4] WHO Effectiveness of interventions to address HIV in prisons Evidence for action technical papers 2007 Geneva: WHO-UNODC-UNAIDS

[B5] Fazel S Bains P Doll H Substance abuse and dependence in prisoners: a systematic review Addiction 2006 101 2 181 191 10.1111/j.1360-0443.2006.01316.x 16445547

[B6] Falissard B Loze JY Gasquet I Duburc A de Beaurepaire C Fagnani F Rouillon F Prevalence of mental disorders in French prisons for men BMC Psychiatry 2006 6 33 10.1186/1471-244X-6-33 16923177 PMC1559686

[B7] Fazel S Danesh J Serious mental disorder in 23000 prisoners: a systematic review of 62 surveys Lancet 2002 359 9306 545 550 10.1016/S0140-6736(02)07740-1 11867106

[B8] Knapp A A retro disease on the loose. Rise of hepatitis C in prisons may fuel outbreak in general population Mod Healthc 2005 35 17 34. 15876002

[B9] Timbart O Les condamnations en 2008 Secrétariat Général, Service support et moyens du ministère, Sous-direction de la Statistique et des Études 2009 Paris: Ministère de la Justice21497357

[B10] Ministry of Health/Justice Guide méthodologique relatif à la prise en charge sanitaire des personnes détenues 2004 Paris

[B11] Ministry of Health/Justice Circulaire n°739, 5 December 1996: Measures to fight against AIDS in prison setting DGS/DH/DAP 1996

[B12] Michel L Carrieri MP Wodak A Harm reduction and equity of access to care for French prisoners: a review Harm Reduct J 2008 5 17 10.1186/1477-7517-5-17 18495018 PMC2430551

[B13] Carrieri MP Rey D Michel L Universal hepatitis B virus vaccination in French prisons: breaking down the last barriers Addiction 2010 105 7 1311 1312 10.1111/j.1360-0443.2010.02968.x 20642514

[B14] Backmund M Meyer K Schuetz C Reimer J Factors associated with exposure to hepatitis B virus in injection drug users Drug Alcohol Depend 2006 84 2 154 159 10.1016/j.drugalcdep.2006.01.009 16481128

[B15] Small W Kain S Laliberte N Schechter MT O'Shaughnessy MV Spittal PM Incarceration, addiction and harm reduction: inmates experience injecting drugs in prison Subst Use Misuse 2005 40 6 831 843 10.1081/JA-200030795 15974143

[B16] Correctional Service Canada Evaluation of HIV/AIDS Harm Reduction Measures in the Correctional Service of Canada 1999 Ottawa: CSC

[B17] Dolan K Shearer J Hall WD Wodak A Bleach is easier to obtain but inmates are still at risk of infection in New South Wales prisons NDARC Technical Report 34 1996 Sydney: National Drug and Alcohol Research Centre, University of New South Wales

[B18] Dolan K Wodak A Hall W HIV risk behavior and prevention in prison: a bleach program for inmates in NSW Drug Alcohol Review 1999 18 139 143 10.1080/09595239996563

[B19] MacDonald M A Study of health care provision, existing drug services and strategies operating in prisons in ten countries from Central and Eastern Europe 2005 Helsinki: The European Institute for Crime Prevention and Control. HEUNI

[B20] Dolan K Prison research Addiction 2009 104 2 223. 19149816 10.1111/j.1360-0443.2008.02489.x

[B21] Jauffret-Roustide M Le Strat Y Couturier E Thierry D Rondy M Quaglia M Razafandratsima N Emmanuelli J Guibert G Barin F Desenclos JC A national cross-sectional study among drug-users in France: Epidemiology of HCV and highlight on practical and statistical aspects of the design BMC Infect Dis 2009 9 1 113. 10.1186/1471-2334-9-113 19607712 PMC2733898

[B22] Greenwald G Drug Decriminalization in Portugal: Lessons for Creating Fair and Successful Drug Policies 2009 Washington, D.C.: CATO Institute

