# Supplementary material for: Limited access to HIV prevention in French prisons (ANRS PRI2DE): implications for public health and drug policy
Source: BMC Public Health. 2011 May 27;11:400. doi: 10.1186/1471-2458-11-400 (PMC3128573; doi:10.1186/1471-2458-11-400)
Supplement: Additional file 1 — ANRS-PRI2 DE Inventory Questionnaire. This file presents the questionnaire, collecting data about characteristics of the prisons with 10 sections, accounting for a total of 46 items, each exploring access to a specific prevention measure. [file 1471-2458-11-400-S1.DOC]

**Questionnaire**

| *Cadre réservé*  *à l’équipe de*  *recherche* |  |
| --- | --- |

**Date of the questionnaire : __________ /__________ / __________**

**Prison Identification**

| **• City :** |  |
| --- | --- |

| **• Prison type (or quarter)** | RC 🞎  PS 🞎  SPS 🞎 | Male 🞎  Female 🞎 | Juvenile 🞎 |
| --- | --- | --- | --- |

| **• Number of prisoners on the day of this study :** |  |
| --- | --- |

| **• Total number of entrant prisoners in 2008 :** |  |
| --- | --- |

**• Sanitary inspection report (2006-2009 program) availability ?** 🞎 yes 🞎 no

**MEDICAL UNIT**

| **• Name of the head of the medical unit :** |  |
| --- | --- |

| **• Telephone :** |  |
| --- | --- |

| **• E-mail :** |  |
| --- | --- |

** If the OST section of the questionnaire is not filled in , or is only partly filled in , by the head of the medical unit, please identify the organization in charge and a contact:**

| - Organization: |  |
| --- | --- |
| - Name : |  |
| - Function : |  |
| - Phone number : |  |

**1. Bleach**

Information

| Is any information on the use of bleach for infectious harm reduction purpose (injection practices, tattoo, piercing) available ? | yes 🔾 no 🔾 dn 🔾 |
| --- | --- |
| *If yes*, are the instructions of use and concerning preservation conditions intelligible for all the prisoners ? | yes 🔾 no 🔾 dn 🔾 |
|  In your opinion, are the prisoners informed of their free access right to bleach every 2 weeks? | yes 🔾 no 🔾 dn 🔾 |

**Quality**

|  Do you know the titration of bleach ? | yes 🔾 no 🔾 dn 🔾 | *if yes* | 3,6° 🔾 12° 🔾 |
| --- | --- | --- | --- |
|  Do you supervise bleach titration? | yes 🔾 no 🔾 dn 🔾 |  | |
|  Do you supervise bleach use- by-date? | yes 🔾 no 🔾 dn 🔾 |  | |

**Access**

|  Is the bleach renewal systematic ? | yes 🔾 no 🔾 dn 🔾 |
| --- | --- |

| *If yes*, at what frequency ? | 🞎 | Every 2 weeks | |
| --- | --- | --- | --- |
|  | 🞎 | If different, please detail*:* |  |

|  Is the bleach distributed on prisoner request? | yes 🔾 no 🔾 dn 🔾 |
| --- | --- |
|  Is bleach buyable inside prison? | yes 🔾 no 🔾 dn 🔾 |
|  Is bleach available in the medical unit ? | yes 🔾 no 🔾 dn 🔾 |

**2. Condoms and Lubricants**

Information

|  In your opinion, are the prisoners informed about their right of access to condoms and the way to use them ? | yes 🔾 no 🔾 dn 🔾 |
| --- | --- |

**Access**

|  Are the following tools accessible inside prison ? | **Male condoms** | **Female condoms** | **Lubricants** |
| --- | --- | --- | --- |
|  | yes 🔾 no 🔾 dn 🔾 | yes 🔾 no 🔾 dn 🔾 | yes 🔾 no 🔾 dn 🔾 |

|  *If yes,* is it **at entry** : | | **Male condoms** | **Female condoms** | **Lubricants** |
| --- | --- | --- | --- | --- |
| Inside the entrant package | | 🞎 | 🞎 | 🞎 |
| During the “entrant” medical consultation | | 🞎 | 🞎 | 🞎 |
| Other | | 🞎 | 🞎 | 🞎 |
| *Please detail* : |  | | | |

|  *If yes,* during **incarceration are they** : | | **Male condoms** | **Female condoms** | **Lubricants** |
| --- | --- | --- | --- | --- |
| In the medical unit | | 🞎 | 🞎 | 🞎 |
| Dispenser inside prison | | 🞎 | 🞎 | 🞎 |
| NGO’s | | 🞎 | 🞎 | 🞎 |
| Social workers | | 🞎 | 🞎 | 🞎 |
| Buyable | | 🞎 | 🞎 | 🞎 |
| Visiting room | | 🞎 | 🞎 | 🞎 |
| Library | | 🞎 | 🞎 | 🞎 |
| Release package (parole) | | 🞎 | 🞎 | 🞎 |
| Other, | | 🞎 | 🞎 | 🞎 |
| *Please detail* : |  | | | |

**3. Opioid Substitution Therapy (OST)**

 In **June 2009**, how many patients were treated with :

| • Buprenorphine : |  |
| --- | --- |

| • Methadone : |  |
| --- | --- |

| • Other OST : |  | *Which one ?* :……………………………………………………………… |
| --- | --- | --- |

**a) OST prescribing**

|  Who is in charge of OST prescribing ? | Medical Unit 🞎 | Psychiatric Unit 🞎 | Addictologic  Unit 🞎 |
| --- | --- | --- | --- |

• Prescription **at entry**

*1^st^ situation : prisoner without OST at entry*

|  Do you initiate methadone treatment ? | yes 🔾 no 🔾 |
| --- | --- |
|  Do you initiate buprenorphine treatment ? | yes 🔾 no 🔾 |
|  Do you initiate another OST ? | yes 🔾 no 🔾 |

*2^nd^ situation : prisoner with OST at entry*

|  Do you continue the same treatment inside prison ? | yes 🔾 no 🔾 |
| --- | --- |

*If you continue with the same treatment,*

|  Do you look for a prescription confirmation (previous physician prescription) ? | yes 🔾 no 🔾 |
| --- | --- |
|  Do you practice a clinical examination as for an OST initiation ? | yes 🔾 no 🔾 |

*If you stop the treatment,*

|  Do you switch to another OST ? | | yes 🔾 no 🔾 |  |
| --- | --- | --- | --- |
|  Except in the case of patient asking for OST withdrawal, do you stop OST ? | | yes 🔾 no 🔾 |  |
| *If yes,*  For what reasons ? |  | | |

** Prescription during incarceration**

|  Do you initiate methadone ? | yes 🔾 no 🔾 |
| --- | --- |
|  Do you initiate buprenorphine ? | yes 🔾 no 🔾 |
|  Do you initiate another OST ? | yes 🔾 no 🔾 |

|  When you initiate OST, do you have a first line OST ? | | yes 🔾 no 🔾 |
| --- | --- | --- |
| *If yes*,  Which one and for what reasons ? |  | |

**b) OST Dosage**

Do you practice ceiling dosages :

|  for methadone ? | | yes 🔾 no 🔾 |  |
| --- | --- | --- | --- |
|  for buprenorphine ? | | yes 🔾 no 🔾 |  |
|  for other OSTs ? | | yes 🔾 no 🔾 |  |
| *If yes*,  For what reasons ? |  | | |

**c) OST distribution**

|  Who is responsible for OST distribution ? | Medical Unit 🞎 | Psychiatric Unit 🞎 | Addictologic  Unit 🞎 |
| --- | --- | --- | --- |

| ***Place of distribution*** | **Methadone** | **Buprenorphine** | **Other OST** |
| --- | --- | --- | --- |
|  in cell ? | yes 🔾 no 🔾 | yes 🔾 no 🔾 | yes 🔾 no 🔾 |
|  in a sanitary unit ? | yes 🔾 no 🔾 | yes 🔾 no 🔾 | yes 🔾 no 🔾 |

| ***Distribution frequency*** | | **Methadone** | **Buprenorphine** | **Other OST** |
| --- | --- | --- | --- | --- |
|  Do you apply a “daily distribution” principle ? | | yes 🔾 no 🔾 | yes 🔾 no 🔾 | yes 🔾 no 🔾 |
|  Is a distribution for several days possible for some patients or situations? | | yes 🔾 no 🔾 | yes 🔾 no 🔾 | yes 🔾 no 🔾 |
| *If yes*,  Please detail |  | | | |

|  Are buprenorphine tablets : | **Crushed ?** | **Diluted ?** |
| --- | --- | --- |
|  | yes 🔾 no 🔾 | yes 🔾 no 🔾 |

**4. Information on HIV, STI, hepatitis and harm reduction (HR)**

|  Are some specific information tools on HIV, STI, hepatitis and HR available for prisoners **at prison entry** (flyers, collective sessions, etc.) ? | | | | yes 🔾 no 🔾 dn 🔾 | |  |
| --- | --- | --- | --- | --- | --- | --- |
|  Are some specific information tools available for prisoners **during incarceration** ? | | | | yes 🔾 no 🔾 dn 🔾 | |  |
|  Do you organize information sessions by external clinical staff (NGO, …) on these topics? | | | | yes 🔾 no 🔾 dn 🔾 | |  |
| *If yes*,  Do they consist in : | 🞎 | Individual counselling ? |  | | | |
|  | 🞎 | Collective counselling ? |  | | | |
|  Do you provide health education programs on HIV, STI, hepatitis and HR, etc. ? | | | | | yes 🔾 no 🔾 dn 🔾 | |

**5. Possible other harm reduction tools**

|  To your knowledge, are some other supplementary HR tools available in the community also available in your prison | | |
| --- | --- | --- |
| 🞎 | Alcohol pads | |
| 🞎 | Sterile water doses | |
| 🞎 | Sterile filters | |
| 🞎 | Sterile cupule | |
| 🞎 | Syringes | |
| 🞎 | Straw | |
| 🞎 | Dressing | |
| 🞎 | Healing cream | |
| 🞎 | Other, *please detail* : |  |

**6. Tools or protocols related to other practices with risk of infection**

|  Is there any standardized protocol to prevent BBV infections transmitted through unsafe hair-cutting procedures ? | yes 🔾 no 🔾 dn 🔾 |
| --- | --- |
|  Is there any protocol or measure concerning hygiene tool access (razors , nail cutters and other sharp hygiene tools) ? | yes 🔾 no 🔾 dn 🔾 |
|  Is there any protocol concerning other risky practices (tattoo, piercing, ...) ? | yes 🔾 no 🔾 dn 🔾 |

Any comment ?

**7. HIV and Hepatitis testing**

| ***A entry*** | **HIV** | **HBV** | **HCV** |
| --- | --- | --- | --- |
|  Is testing systematically proposed ? | yes 🔾 no 🔾 | yes 🔾 no 🔾 | yes 🔾 no 🔾 |
|  Are negative results systematically returned to prisoners ? | yes 🔾 no 🔾 | yes 🔾 no 🔾 | yes 🔾 no 🔾 |

*Except in the case where testing was not done at prison entry and is proposed at a later date, is it possible to renew the testing* ***later during incarceration?*** *yes  no *

| *If yes, is it :* | | |  |  | | |
| --- | --- | --- | --- | --- | --- | --- |
|  following medical unit proposal ? | | | yes 🔾 no 🔾 |  | | |
|  following prisoner request ? | | | yes 🔾 no 🔾 |  | | |
|  When testing is performed during incarceration, what are the reasons considered ? | | | | | |  |
| 🞎 | Risky sexual behaviour | | | |  | |
| 🞎 | Risky drug related behaviour | | | |  | |
| 🞎 | Other risky behaviours | | | |  | |
|  | *If yes,* please detail ? |  | | | | |

|  Is an HBV vaccination systematically proposed to HBV negative prisoners? | yes 🔾 no 🔾 |
| --- | --- |

**8. STI screening**

| ***At prison entry*** |  |
| --- | --- |
|  Is a screening systematically proposed for the following STI : |  |
| Chlamydia | yes 🔾 no 🔾 |
| Gonococcus | yes 🔾 no 🔾 |
| Syphilis | yes 🔾 no 🔾 |
| Herpes | yes 🔾 no 🔾 |
|  Are negative results systematically returned to prisoners ? | yes 🔾 no 🔾 |

| ***During incarceration*** |  |
| --- | --- |
|  Is the STI screening proposed following a medical unit proposal ? | yes 🔾 no 🔾 |
|  Is the STI screening proposed following a prisoner proposal ? | yes 🔾 no 🔾 |
|  Did the medical unit treat STI acquired **during incarceration** ? | yes 🔾 no 🔾 |

**9. Post-Exposition Prophylaxis (PEP )**

|  Does the PEP disposal include written recommendations related to the 13 March 2008 circular ? | | | yes 🔾 no 🔾 |
| --- | --- | --- | --- |
|  Are the prisoners aware of the PEP availability inside the prison ? | | | yes 🔾 no 🔾 dn 🔾 |
|  Are the security guards aware of the PEP availability inside the prison ? | | | yes 🔾 no 🔾 dn 🔾 |
|  Did the medical unit prescribe a PEP during the last 12 months ? | | | yes 🔾 no 🔾 |
| *If yes*, | | |  |
| How many PEP ? |  |  | |

| For what reason ? | 🞎 | Sexual exposure | |  |
| --- | --- | --- | --- | --- |
|  | 🞎 | Exposure related to drug use | |  |
|  | 🞎 | Other situation | |  |
| *If other, please detail* : | | |  | |

**10. Risk indicators related cares inside prison**

|  Did you provide cares for abscesses potentially associated to injection? | yes 🔾 no 🔾 |
| --- | --- |
|  Did you provide cares for other complications potentially associated to injection? | yes 🔾 no 🔾 |
|  Did you provide cares for skin infections potentially associated to tattooing and other scarring practices? | yes 🔾 no 🔾 |
|  Did you provide care for nose bleeding potentially associated to sniffing practices ? | yes 🔾 no 🔾 |

**11. Would you like to provide complementary information related to the topics explored in this questionnaire?**

** Would you please propose us the name of a nurse for a further phone interview**

| - Name : | ____________________________________________________________________ |
| --- | --- |
| - Role : | ____________________________________________________________________ |
| - Phone number : | ____________________________________________________________________ |
| - E-Mail : | ____________________________________________________________________ |
| - Seniority in the prison | ____________________________________________________________________ |

**Questionnaire à renvoyer :**

- **Par courrier postal à :**

**ANRS**

**Service Recherches en Santé publique & Sciences de l’homme et de la société**

**Projet PRI^2^DE**

**101, rue de Tolbiac – 75013 Paris**

- **Par courrier électronique à :ccmo@ccmoconseil.com**
